# Profiles of Multidrug Resistance Protein-1 in the Peripheral Blood Mononuclear Cells of Patients with Refractory Epilepsy

**DOI:** 10.1371/journal.pone.0036985

**Published:** 2012-05-14

**Authors:** Jae-Jun Ban, Keun-Hwa Jung, Kon Chu, Soon-Tae Lee, Daejong Jeon, Kyung-Il Park, Hye-Jin Moon, Hyeyun Kim, Sunghun Kim, Sang Kun Lee, Jae-Kyu Roh

**Affiliations:** 1 Department of Neurology, Laboratory for Neurotherapeutics, Seoul National University Hospital, Seoul, South Korea; 2 Program in Neuroscience, Neuroscience Research Institute of SNUMRC, Seoul National University, Seoul, South Korea; 3 Comprehensive Epilepsy Center, Seoul National University Hospital, Seoul, South Korea; 4 Department of Bio and Brain Engineering, Korea Advanced Institute of Science and Technology (KAIST), Daejeon, South Korea; 5 Departments of Neurology, Inje University Baik Hospital, Seoul, South Korea; 6 Kwandong University Myongji Hospital, Goyang, South Korea; 7 Gangwon National University College of Medicine, Chuncheon, South Korea; University of California San Francisco, United States of America

## Abstract

**Background:**

About one third of patients with epilepsy become refractory to therapy despite receiving adequate medical treatment, possibly from multidrug resistance. P-glycoprotein, encoded by multidrug resistance protein-1 (MDR1) gene, at the blood brain barrier is considered as a major factor mediating drug efflux and contributing to resistance. Given that peripheral blood mononuclear cells (PBMNCs) express MDR1, we investigated a MDR1 status of PBMNCs in various subsets of epilepsy patients and demonstrated their association with clinical characteristics.

**Methodology/Principal Findings:**

Clinical and MDR1 data were collected from 140 patients with epilepsy, 30 healthy volunteers, and 20 control patients taking anti-epileptic drugs. PBMNCs were isolated, and basal MDR1 levels and MDR1 conformational change levels were measured by flow cytometry. MDR1 profiles were analyzed according to various clinical parameters, including seizure frequency and number of medications used in epilepsy patients. Epilepsy patients had a higher basal MDR1 level than non-epilepsy groups (p<0.01). Among epilepsy patients, there is a tendency for higher seizure frequency group to have higher basal MDR1 level (p = 0.059). The MDR1 conformational change level was significantly higher in the high-medication-use group than the low-use group (p = 0.028). Basal MDR1 (OR = 1.16 [95% CI: 1.060–1.268]) and conformational change level (OR = 1.11 [95% CI: 1.02–1.20]) were independent predictors for seizure frequency and number of medications, respectively.

**Conclusions/Significance:**

The MDR1 profile of PBMNCs is associated with seizure frequency and medication conditions in patients with epilepsy.

## Introduction

Almost one-third of patients with epilepsy cannot achieve seizure freedom and have a poor prognosis despite receiving adequate medical treatment and the administration of multiple anti-epileptic drugs (AEDs) [1]. Although the mechanism of this refractoriness remains unclear, the prediction of medically refractory epilepsy is an important concern of current epilepsy research.

Multidrug resistance protein-1 (MDR1), multidrug resistance-related proteins and breast cancer resistance proteins comprise members of the multidrug resistance protein family. MDR1, first known as a drug-resistance protein in tumor cells [2], is a 170- to 180-kDa membrane protein. MDR1 mediates ATP-dependent drug efflux for various xenobiotics and drugs [3], [4]._ENREF_2 The apical membranes of capillary endothelial cells of the blood-brain barrier express MDR1, which contributes to drug efflux from the brain to the systemic circulation. Despite the protective role of transporter proteins, AED penetration to the brain is compromised by MDR1 [5]–[7]. For intractable patients with epilepsy, drug resistance is likely caused by a high level of MDR1 in the brain endothelium [8]–[10].

Recent studies suggest that molecular profile of peripheral blood mononuclear cells (PBMNCs) could be used as biopsy material to reflect physiological condition of the body and a possible marker for predicting the treatment outcome of various diseases [11]–[17]. Given that human peripheral blood cells also express MDR1 proteins and play a role in brain endothelial cell turnover [18], we hypothesized that the MDR1 profile of these cells might be related to drug resistance of epilepsy.

In this study, we examined epilepsy patients, healthy volunteers, and non-epilepsy patients taking AEDs. We also divided refractory epilepsy patients according to their seizure frequency and the number of medications required to control their seizures, which could be regarded as criteria of refractoriness of epilepsy [1], [19]. For MDR1 status, we examined basal MDR1 level and conformational change level of PBMNCs by flow cytometry. Both the basal MDR1 level and conformational change level were measured using the UIC2 antibody, which binds to the MDR1 protein and has high affinity to the activated ATP-hydrolyzing form of MDR1 in the presence of substrate [20], [21]. The enhanced affinity of this antibody allows for detection of cells with relatively low MDR1 expression. The basal MDR1 level, which was analyzed by a conventional flow cytometry after staining unstimulated PBMNCs, reflects the number of cells expressing high level of MDR1 protein. The MDR1 conformational change level measured in vinblastine-treated PBMNCs, which have activated MDR1, reflects the number of all cells with functional MDR1 which has the ability of actual pumping action in peripheral blood [22], [23]. Several clinical parameters were assessed in association with the categorized MDR1 data.

**Figure 1:**
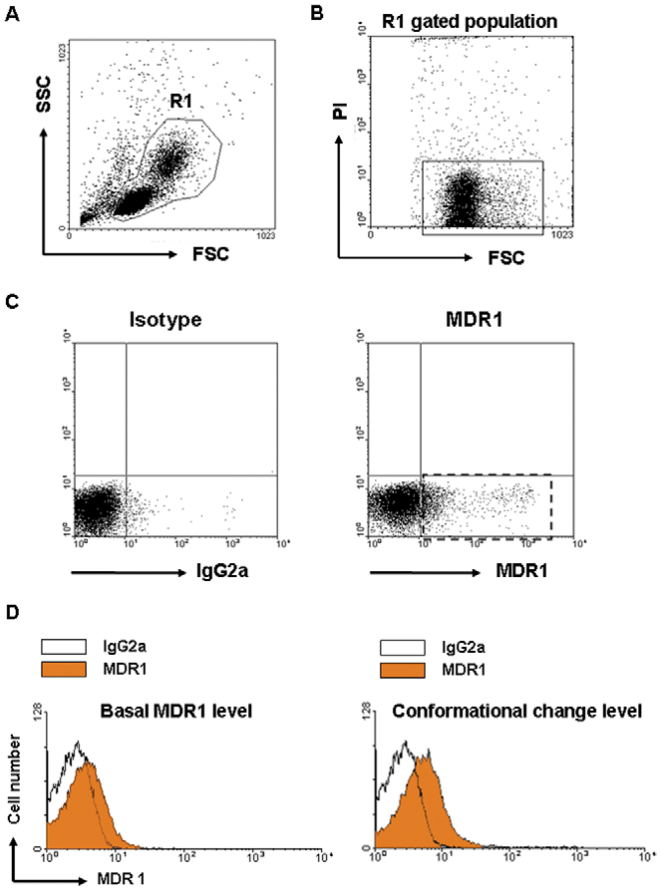
Analysis of MDR1 profiles of PBMNCs with the flow cytometry. (A) PBMNCs were stained with MDR1 antibody and propidium iodide (PI) and examined forward scatter (FSC) and side scatter (SSC) and leukocytes were gated (R1) to exclude aggregates and debris. (B) In R1 gated population, PI positive dead cells were excluded and PI negative live cells were gated (lined box). (C) In gated cells, the MDR1-positive population was assessed using the baseline determined from a negative control of IgG2a background staining (dotted box). (D) Representative histogram figures show the basal MDR1 level (left) and conformational change level (right).

**Table 1:** Refractory epilepsy patient demographics.

Parameters	Refractory epilepsy (n = 117)
Age (year)	37.8±10.8
Age of onset (year)	20.3±13.4
BMI	35.2±8.1
Hypertension (n, %)	10 (8.5)
Diabetes (n, %)	3 (2.6)
Hyperlipidemia (n, %)	1 (1)
Duration of epilepsy (year)	18.8±9.2
Frequencies of seizures (per week)	1.7±4.5
Seizure duration (sec)	110±162.9
Temporal lobectomy (n, %)	10 (8.5)
Smoking (n, %)	14 (12)
Febrile seizure (n, %)	19 (16.2)
GTCS : CPS : SPS (%)	57.1∶ 25 : 17.9
Family history (n, %)	8 (6.8)
Number of AEDs	2.8±1.3

GTCS = generalized tonic-clonic seizure; CPS = complex partial seizure; SPS = simple partial seizure.

## Materials and Methods

### Ethics Statement

The study protocol was approved by the Scientific Review Committee and the Institutional Review Board of Seoul National University Hospital, and all enrolled subjects provided written informed consent as required by the Declaration of Helsinki.

### Study Population

We studied 117 refractory and 23 seizure-free epilepsy patients who were diagnosed with temporal lobe epilepsy, and 20 control patients taking AEDs. The seizure-free epilepsy patients didn’t experience seizure for at least one year. The control with AEDs consisted of non-epilepsy patients taking AEDs because of neuropathic pain, headache and etc. The patients underwent medical follow-up at the Department of Neurology, Seoul National University Hospital, from February 2008 to December 2010. Thirty healthy volunteers were recruited for the control group. A questionnaire was developed by the authors to evaluate the patients’ demographic data and medical histories.

**Table 2:** Control groups characteristics.

Parameters	Healthy control	Non-epilepsy with AEDs	Seizure-free epilepsy
	(n = 30)	(n = 20)	(n = 23)
Age (year)	37.8±10.8	49.9±13.6	32.3±10.7
Number of AEDs	0	1.1±0.3	1.3±0.4
Type of AEDs (n)	–	VPA(15), PRE(2),	VPA(3), CBZ(2), OCZ(5),
		TPM(1), VPA+PRE(2)	LEV(1), TPM(2), LTG(2),
			CBZ+LTG(1), CBZ+LEV(1),
			LEV+LTG(1), CBZ+CLO(1),
			TPM+CLO(1), DPH+PB(1),
			LEV+TPM(1), CBZ+TPM(1)

VPA = valproate; CBZ = carbamazepine; OCZ = oxcarbazepine; LEV = levetiracetam; TPM = topiramate; LTG = lamotrigine; CLO = clobazam; PB = phenobarbital; PRE = pregabalin.

**Figure 2:**
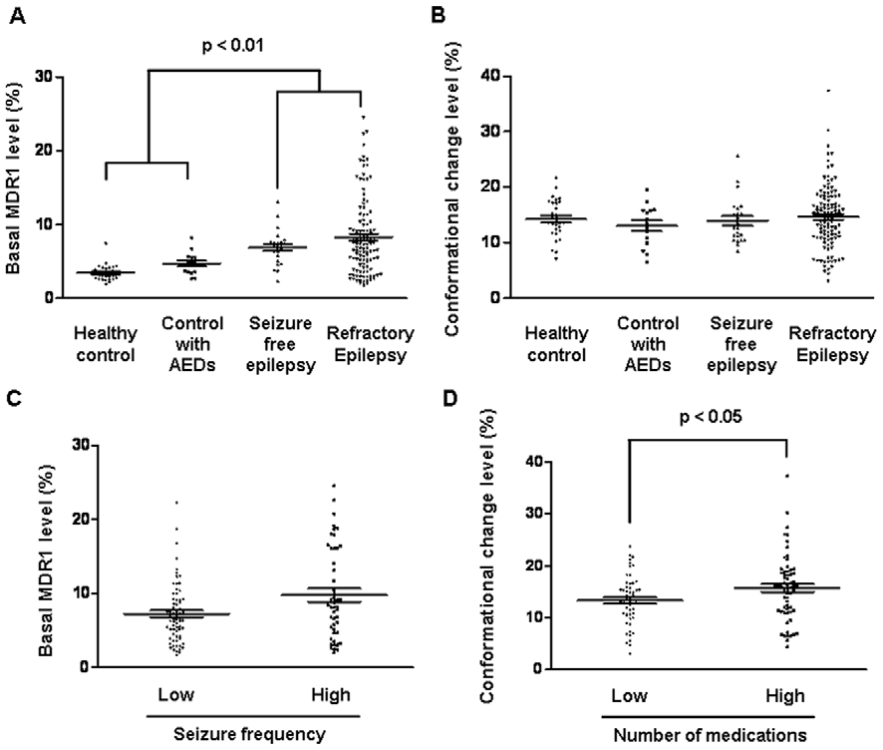
MDR1 profiles among groups and refractory epilepsy patients. Graphs represent (A) basal MDR1 level and (B) conformational change level among the groups. Graphs represent (C) basal MDR1 level between low- and high-seizure-frequency groups and (D) conformational change level between low- and high-medication-use groups in the refractory epilepsy patients. The horizontal lines represent the median level and standard error of the mean.

### PBMNC Preparation and Flow-cytometric Analysis

Materials for the flow cytometry staining including MDR1 antibody (UIC2 antibody), dimethyl sulfoxide (DMSO), vinblastine, working solutions, and binding buffers were all included in the MDR1 shift assay kit (Chemicon International, Temecula, CA, USA). Peripheral blood samples (5cc) were obtained from all enrolled subjects. The heparinized blood samples were added to Histopaque (Sigma-Aldrich, St. Louis, MO, USA) and centrifuged at 400 ×g for 30 min to separate the PBMNCs. Isolated cells were counted and centrifuged at 200 ×g for 5 min. The supernatant was removed, and the pellets were resuspended to 1×10^6^ cells/mL in warmed UIC2 binding buffer (1% bovine serum albumin in PBS). Basal MDR1 level assay procedure is as follows: cells were divided into two groups and treated with 5 µL of DMSO respectively. The tubes were incubated at 37°C for 10 min; 25 µL of the kit’s IgG2a antibody working solution was added to one group and 25 µL of UIC2 antibody working solution was added to the other group. The tubes were incubated at 37°C for another 15 min and washed three times with cold UIC2 binding buffer at 200×g for 10 min at 4°C. The cells were resuspended in 250 µL ice-cold secondary antibody working solution and incubated for 15 min at 4°C in the dark. After being washed three times, the cells were resuspended in 0.5 mL ice-cold propidium iodide staining buffer. Samples were then maintained on ice until analysis with a flow cytometer (FACSCalibur, BD Biosciences, San Jose, CA, USA), using FL2 channel for indirect UIC2 staining and FL3 channel for propidium iodide to exclude dead cells. To filter out cell debris, forward scatter and side scatter of the cells were measured and lymphocytes and monocytes regions were gated (Fig. 1A). After gating cell populations, propidium iodide negative cells were gated to wipe out dead cells (Fig. 1B). Using this cell population, baseline for gating positive population was confirmed using IgG2a isotype. UIC2 positive populations were gated using this baseline and positive cell number was counted using FL2 channel (Fig. 1C). Positive level was calculated as a percentage of UIC2 positive cell number in whole live cell population. For MDR1 conformational change level assay, we used 5 µL of 22 mM vinblastine instead of DMSO and rest of the process is identical to basal MDR1 level. UIC2 antibody reacts with membrane bound MDR1 and their reactivity is increased when conformational change of MDR1 occurs. The basal MDR1 level reflects the number of cells expressing high level of MDR1 and the conformational change level reflects all functional MDR1 level in PBMNCs [22], [23].

### Statistical Analysis

Data were analyzed using SPSS version 17.0 (SPSS Inc., Chicago, IL, USA). Student’s t-test or analysis of variance with subsequent Tukey’s b-test for MDR1 conformational change level, Mann-whitheny U test or Kruskal-Wallis test for basal MDR1 level were used to compare mean values for continuous variables, and the chi-squared test was used for categorical variables. We classified refractory epilepsy patients in terms of seizure frequency. The low-frequency group had fewer than one seizure per week (n = 69, 59%) and the high-frequency group had one or more seizures per week (n = 48, 41%). We also classified the patients into those taking three or more medications (n = 63, 54%) and those taking fewer than three (n = 54, 46%). To identify factors helping to predict high seizure frequency and medication number, multiple logistic regression analysis was performed with significant variables defined from the initial univariate analysis, and statistical significance was set at p<0.05.

**Table 3:** Correlation between seizure frequency and clinical parameters.

	Seizure frequency		
Parameters	Low (n = 69)	High (n = 48)	*p* Value
Frequency of seizure (per week)	0.1±0.2	4±6.5	<0.001
Age (year)	38±11	37.5±10.7	0.817
Age of onset (year)	20.8±13.2	19.5±13.7	0.632
Duration of epilepsy (year)	17±11.8	17.7±10	0.752
Number of AEDs	2.6±1.2	3.2±1.4	0.015*
Seizure duration (sec)	118.8±172.0	98.6±150.4	0.515
BMI	35.8±7.6	34.3±8.9	0.323
Hypertension: n (%)	5 (7.2)	5 (10.4)	0.738
Diabetes: n (%)	0 (0)	3 (6.3)	0.066
Hyperlipidemia: n (%)	0 (0)	1 (2.1)	0.410
Smoking: n (%)	7 (10.1)	7 (14.6)	0.467
Temporal lobectomy: n (%)	3 (4.3)	7 (14.6)	0.089
Febrile seizure: n (%)	16 (23.2)	3 (6.3)	0.015*
Family history: n (%)	4 (5.8)	4 (8.3)	0.715
Semiology: (GTCS: CPS: SPS,%)	58.2∶ 23.9∶ 17.9	55.6∶ 26.7∶ 17.8	0.943
Basal MDR1 level (%)	7.2±4.0	9.7±6.2	0.009**
Conformational change level (%)	14.2±5.2	14.9±6.0	0.512

BMI = body mass index.

* p<0.05,

** p<0.01.

**Table 4:** Multiple logistic regression analysis of factors for the seizure frequency and medication number.

Seizure frequency			Medication number		
Factors	Odd ratio (95% CI)	*p* Value	Factors	Odd ratio (95% CI)	*p* Value
Temporal lobectomy	12.093 (2.197–66.55)	0.004	Epilepsy duration	1.06 (1–1.116)	0.017
Medication number	2.751 (1.161–6.521)	0.022	Onset age	0.99 (0.99–1)	0.838
Basal MDR1 level	1.16 (1.060–1.268)	0.001	MDR1 conformational change	1.11 (1.02–1.2)	0.008

CI = Confidence interval.

## Results

### Patient Characteristics

The study was carried out in 190 subjects, including 117 refractory epilepsy patients, 23 seizure-free epilepsy patients, 30 healthy volunteers, and 20 control patients taking AEDs. The baseline characteristics of the refractory epilepsy patients are summarized in Table 1. Characteristics, including age, number of AEDs and type of AEDs, of control groups are represented in Table 2.

### MDR1 Profiles Among Patients

The basal MDR1 and conformational change level of MDR1 in the PBMNCs were analyzed using flow cytometry. Representative superimposed histograms of basal MDR1 level and conformational change level are presented (Fig. 1D). Kruskal-Wallis test confirmed significant differences in the basal MDR1 levels among groups (p<0.01). Patients with epilepsy had higher basal MDR1 levels than the healthy volunteers and control patients taking AEDs (p<0.01; Fig. 2A). The conformational change level was not significantly different among groups (Fig. 2B).

### MDR1 Profiles According to Seizure Frequency

We classified refractory patients into high- and low-seizure-frequency groups (Table 3). The high-frequency group took more medications (3.2±1.4 vs. 2.6±1.7, p = 0.015) than the low-frequency group. The high-frequency group tended to have a higher basal MDR1 level (9.7±6.2% vs. 7.2±4.0%, p = 0.059) (Fig. 2C). Moreover, independent associations with seizure frequency were noted for basal MDR1 level (p = 0.001), number of medications (p = 0.022), and a history of temporal lobectomy (p = 0.004). The odds ratio of the basal MDR1 level for seizure frequency was 1.160 (95% CI, 1.060–1.268) after adjustment (Table 4).

### MDR1 Profiles According to AED Intake

To evaluate the association between the MDR1 profile and drug resistance, we categorized patients into high- and low-medication-use groups. All variables and statistical data are shown in Table 5. The high-medication group had a younger age of onset and a longer duration of epilepsy (17.6±13.2 vs. 23.3±13.1 years, p = 0.02; 20.6±10.8 vs. 13.5±10 years, p<0.05) (Fig. 2D). The mean seizure frequency tended to be higher in the high-medication group, but it was not significant. In terms of MDR1 status, we found a higher conformational change level in the high-medication group than in the low-medication group (p = 0.028), whereas basal MDR1 level was not significantly different between these groups. Multiple logistic regression analysis indicated that the duration of epilepsy and the conformational change level were significant independent predictors of the number of AEDs taken (Table 4). The odds ratio of conformational change level of MDR1 for medication number was 1.11 (95% CI, 1.02–1.20).

**Table 5:** Correlation between medication number and clinical parameters.

	Medication number		
Parameters	Low (n = 54)	High (n = 63)	*p* Value
Number of AEDs	1.6±0.4	3.9±0.9	<0.001
Age (year)	37.4±11.6	38.2±10.2	0.174
Age of onset (year)	23.3±13.1	17.6±13.2	0.020*
Duration of epilepsy (year)	13.5±10.1	20.6±10.8	<0.001**
Frequency of seizure (per week)	1.2±2.7	2.2±5.7	0.246
Seizure duration (sec)	146.4±200.1	78.9±114.5	0.033*
BMI	34.8±7.7	35.5±8.5	0.650
Hypertension: n (%)	3 (5.6)	7 (11.1)	0.337
Diabetes: n (%)	1 (1.9)	2 (3.2)	1
Hyperlipidemia: n (%)	0 (0)	1 (1.6)	1
Smoking: n (%)	6 (11.1)	8 (12.7)	0.792
Temporal lobectomy: n (%)	4 (7.4)	6 (9.5)	0.751
Febrile seizure: n (%)	7 (13)	12 (19)	0.374
Family history: n (%)	4 (7.4)	4 (6.3)	1
Semiology: (GTCS: CPS: SPS,%)	58.5∶22.6∶18.9	55.9∶27.1∶16.9	0.855
Basal MDR1 level (%)	8.5±5.1	8.0±5.2	0.634
Conformational change level (%)	13.3±4.6	15.7±6.3	0.028*

* p<0.05,

** p<0.01.

## Discussion

We assayed the MDR1 profile of PBMNCs in epilepsy patients by flow cytometry. We categorized 117 patients with refractory epilepsy according to seizure frequency and the number of medications taken. These two parameters could be regarded as criteria for the severity of refractoriness [19], [24]. Basal MDR1 level and conformational change level were higher in the high-seizure frequency group and high-medication group, respectively. Multiple logistic regression analysis revealed these as independently related factors.

Our results suggested that the basal MDR1 level was higher in epilepsy patients compared with healthy volunteers and non-epilepsy patients taking AEDs. The AEDs were administered to non-epilepsy patients for treatment of migraine, neuropathic pain, and etc. The regimens included valproic acid, topiramate or pregabalin, and their dosage ranges were much lower than those in the refractory epilepsy patients. The lower dosage range and different type of anticonvulsants could influence lower MDR1 level of PBMNCs in non-epilepsy with AEDs than epilepsy with AEDs. However, we found no significant difference according to the types of AEDs among epilepsy patients. In this study, the high-seizure-frequency group also took more medications. This group experienced recurrent seizures despite adequate administration of multiple AEDs. Although these drugs control seizures initially, drug resistance may gradually emerge from the overexpression of MDR1, leading to refractory epilepsy [25]. Our result shows that the patients who experience more seizure tend to have more cells expressing high level of MDR1 in peripheral blood. In addition to categorizing patients according to seizure frequency, we examined high- and low-medication groups. The use of more medications indicates an insufficient treatment outcome from a single major AED. This result suggests that the high-medication patients have more cells having functional MDR1 in PBMNCs than low-medication patients.

Our findings suggest that MDR1 profile of PBMNCs is associated with seizure severity and medication conditions in underlying refractory epilepsy patients. The mechanism of these differences remains elucidated. There have been no animal experiments for investigating peripheral MDR1 status in epilepsy. We speculate that prolonged exposure of multiple AEDs could influence individual variations of MDR1 level, since several of these drugs are believed to be substrate or inducer of MDR1 [26], [27]. We classified drugs as well-known MDR1 substrate (phenobarbital, oxcarbazepine and lamotrigine) and non-substrate and analyzed the data. But we couldn’t obtain meaningful differences among drugs in epilepsy patients. Since there are many other variables such as duration of administration of each drug, and controversies about AEDs as MDR1 substrates, further research is needed to confirm this concept. It is also possible that the epilepsy influences profile of population of peripheral blood cells. A severe degree of seizures impair brain cells and this condition can influence some kind of circulating stem cells [18], [28], and alternately, a subset of PBMNCs, i.e., endothelial progenitor cells might involve endothelial cell turnover in the brain of epilepsy patients.

The present findings seem to be consistent with other researches, which indicate MDR1 status is related to refractoriness in epilepsy patient. Ideally, we should investigate the MDR1 status of epilepsy patients in vivo, for example, by using positron emission tomography with radiolabeled AEDs [29], [30]. However, this is costly and time consuming, whereas the MDR1 status of PBMNCs is easily analyzed in one day in the laboratory. Although our study is hampered by study population heterogeneity and low sample size, our approach is the first attempt to seek a potential blood marker associated with a variety of epilepsy phenotypes. We provide novel evidence that the MDR status in PBMNCs can reflect epilepsy phenotype, and it is potentially altered by refractory itself or long term AED medication. A prospective longitudinal study is needed to confirm the predictive value of MDR1 level.
